# Dynamics of soil nitrogen fractions and their relationship with soil microbial communities in two forest species of northern China

**DOI:** 10.1371/journal.pone.0196567

**Published:** 2018-05-24

**Authors:** Dong Liu, Yimei Huang, Hao Yan, Yueli Jiang, Tong Zhao, Shaoshan An

**Affiliations:** 1 Key Laboratory of Plant Nutrition and the Agri-environment in Northwest China, Ministry of Agriculture, College of Resource and Environment Science, Northwest A&F University, Yangling, China; 2 State Key Laboratory of Soil Erosion and Dryland Farming on Loess Plateau, Northwest A&F University, Yangling, China; RMIT University, AUSTRALIA

## Abstract

Microbially-mediated soil N mineralization and transformation are crucial to plant growth. However, changes in soil microbial groups and various N components are not clearly understood. To explore the relationship between soil N components and microbial communities, we conducted an *in-situ* experiment on two typically planted forest species, namely, *Sibirica Apricot* (SA) and *Prunus davidiana Franch* (PdF) by using closed-top polyvinyl chloride tubes. Changes in soil inorganic N, organic N (ON) fractions, and levels of microbial phospholipid fatty acids (PLFAs) were measured bimonthly from April 2012 to April 2013. Microbial PLFAs and the concentrations of easily-available microbial biomass N (MBN; ~60 mg kg^-1^), soluble ON (SON; ~20 mg kg^-1^), and inorganic N were similar between the two soils whereas the ON (~900 mg kg^-1^) and its major part total acid-hydrolyzable N (HTN; ~500 mg kg^-1^), were significantly different (p < 0.05) in most months (5/6 and 4/6; respectively). The canonical correlation analysis of soil N fractions and microbial parameters indicated that the relationship between total PLFAs (total biomass of living cells) and NH_4_^+^-N was the most representative. The relative contributions (indicated by the absolute value of canonical coefficient) of NH_4_^+^-N were the largest, followed by NO_3_^−^-N and MBN. For the HTN component, the relative percentage of hydrolyzable amino acid N and ammonium N decreased markedly in the first half of the year. Canonical variation mainly reflected the relationship between ammonium N and bacterial PLFAs, which were the most sensitive indicators related to soil N changes. The relative contributions of HTN components to the link between soil microbial groups and HTN components were ammonium N > amino acid N > amino sugar N. Observations from our study indicate the sensitivity of soil N mineralization indicators in relation to the temporal variation of soil microbial groups and N fractions.

## Introduction

Nitrogen (N) is one of the most important nutrients in natural and agricultural ecosystems [1]. Approximately 90% of total soil N is composed of soil organic nitrogen N (ON), which plays an important role in N retention and transformation [2,3]. N availability, which is important for the growth of plants, is closely associated with the mineralization of ON and the depolymerization of the N-containing constituents, namely, amino acid and amino sugar [4,5]. Given the diverse origins/resources and complex composition of N components, ON immobilization and mineralization have been neglected until the ON fractions received considerable attention [6–9]. In general, ON consists of acid-insoluble and acid-hydrolyzable parts, and the acid-hydrolyzable components include amino acids, amino sugars, ammonium, and unknown N sub-fractions. The origin and significance of these N fractions in soil N cycling have been addressed extensively [2,10,11]. However, whether inorganic- and ON fractions, such as microbial biomass N (MBN) and SON, exhibit synchronous patterns during soil N transformations and how this process is affected remains unclear.

Both abiotic (such as precipitation/water content and temperature) and biotic factors (such as plants and underlying soil microbial communities and their distribution) can affect soil N transformation. Soil substrate availability, microbial community structure, temperature-dependent biochemical processes, and transportation of substrates and waste products exhibit strong variations based on monthly changes in precipitation/water content of soil [12–14]. In addition, temporal variations in plant growth and soil C and N availability can affect microbial N transformation [15] and microbial activity [16,17] because precipitation/water content is a key-limiting factor regulating primary productivity and soil microbial activity in arid and semi-arid regions. The Loess Plateau is one of the most severely eroded areas in the semi-arid region [18,19]. As a main source of soil nutrient supply, N mineralization is highly important in the Loess Plateau, where soils are largely barren and deprived of rainfall [20]. However, since the late 1990s, plant coverage has increased from 46.9% to 75.5% with visible effects in soil and water conservation after 20 years of revegetation [19]. With this information for improving ecological function in the Loess Plateau, increasing attention is needed to the effects of revegetation on soil N fractions and mineralization processes, given their key role in terrestrial ecosystems [21]. Plant species affect N mineralization rates [22–24]. For instance, in the subtropical area of eastern China, evergreen broadleaf forests exhibit higher N mineralization rates than that of the fir plantations and secondary shrubs [25]. Ren et al. (2011) also reported that in early successional shrub communities and coniferous forests, the concentrations of soil mineral N and net N mineralization were significantly lower than those of late-successional mixed and broadleaf forests [26]. During N mineralization, tree species also affect soil inorganic N supply. For example, nitrate production via oxidation of ON was three times faster in the soil of scots pine (*Pinus sylvestris L*.) than that of pedunculate oak (*Quercus robur L*.) [27]. These results appeared to be highly dependent on species. Therefore, for an overall understanding of N mineralization, the characteristics of different vegetation in the specific research region should be considered (for example the typical area of the Loess Plateau in Southern Ningxia, China).

Soil N transformations are driven by microorganisms [28–30]. Microbial community dynamics, which are closely linked to soil C and N transformations [31–34] respond differently to changes in the dominant vegetation [35] and substrate concentration [36]. Plantation forest soil microbial communities are affected by multiple factors, including forest type, climate, and soil characteristics [37–39]. Smithwick et al. (2005) found that *in-situ* net N mineralization is spatially correlated with microbial community structure [40]. In forest soils, significant positive correlations exist among NO_3_-N immobilization rate, heterotrophic nitrification rate, and fungal biomass [41]. The heterotrophic nitrification and immobilization of NO_3_-N may be important N transformation pathways affecting ecosystem productivity. However, the relationships and interplay between changes in the various N fractions and the microbial community remain unclear.

In the present study, we selected a typical revegetated region, the Chinese Loess Plateau in Southern Ningxia. Within the investigated region, the typical cultivated forests, *Sibirica Apricot* (SA) and *Prunus davidiana Franch* (PdF), were selected as the representative species to study the relationship between soil N fractions and microbial groups during N mineralization processes. A one-year (from April 2012 to April 2013) *in-situ* field incubation experiment was conducted in the two typical forested sites. The objectives of this study were to i) quantify the dynamics of soil N fractions, including the net rate of the ammonification, nitrification, and mineralization, and to identify the variation in the percentage of the hydrolyzable N components at the monthly scale ii) identify changes in the microbial communities accompanied by soil N change, and iii) further explore the relationship between soil N and microbial community as affected by plant species and time. We formed the following hypotheses: i) the influence of plant species on soil ON is stronger than that on inorganic N; ii) the effect of temporal variation on soil N and microbial community is stronger than that of plant species; and iii) changes in soil microbes are closely related to easily available N forms.

## Materials and methods

### Site description

The study area is located in the southern mountains of Ningxia, China, at the Shang-Huang Ecological Station (35°59'–36°03' N, 106°26'–106°30' E; altitude: 1534–1822 m a.s.l.) of Institute of Soil and Water Conservation of the Chinese Academy of Sciences. The local site experiences a monsoon climate with a transition from semi-arid to warm temperate. The mean annual rainfall is approximately 420 mm. The average annual temperature is approximately 6.9°C. According to the soil classification system of the Food and Agriculture Organization of the United Nations (FAO), the loessial soil [42] represents a silty clay loam texture. The main land-use types are artificial grassland (primarily *Medicago sativa L*.), artificial forestland (*Korshinskii* and *Pyrus spp*. *pear*), abandoned land (*Stipa bungeana Trin*., *Thymus mongolicus*, and *Artemisia giraldii Pamp*.), and farmland (Triticum aestivum and Zea mays).

### Experimental design and field incubation

*Sibirica Apricot* (SA) and *Prunus davidiana Franch* (PdF) are typical plants cultivated on the Chinese Loess Plateau to reduce the rate of soil erosion with an average survival rate of ~50% [43]. The experiment was conducted on the planted field of SA and PdF forestland. For controlling soil spatial variability, we selected two sites with a close distance (<1 km) and similar topography (hillside field) and land-use history (abandoned cropland with previous cultivation of wheat (*Triticum aestivuml*). Soils of the study area were all developed from the same loessial parent soil material. The detailed geographical characteristics are shown in the Table 1. Meanwhile, for minimizing plot effects and obtaining representative soil samples, three 10 m × 10 m replicate subplots were established at each plant species site in April 2012.

**Table 1:** Geographic and vegetation characteristics of the sites.

Vegetation type	*Sibirica Apricot*	*Prunus davidiana* Franch
	(SA)	(PdF)
Planting time (y)	2002	2000
Slope aspect (°)	NE32°	NE41°
Slope degree (°)	4	18
Latitude	N35°59′50.61″	N36°00′0.36″
Longitude	E106°28′1.51″	E106°27′53.77″
Elevation (m a.s.l.)	1617	1632
Main companion	*Stipa bungeana Trin*	*Stipa bungeana Trin*.
Species	*Artemisia scoparia*	
Coverage (%)	60	45

Annual *in-situ* net N mineralization was measured by the buried soil core method [44]. Specifically, after removal of surface litter, six polyvinyl chloride (PVC) cylinders (7 cm in diameter and 11 cm in length) were installed in each subplot, at a depth of 10 cm into the soil and an adjacent distance of 50 cm. One soil core was taken for immediate analysis, the remaining intact soil cores were placed into a PVC collar, and then the soil cores were sealed with plastic wraps on the top to minimize evaporation. Absorbent cotton was placed on the bottom to maintain enough ventilation. All of the sealed PVC collars were placed back into soils at a depth of 10 cm. Aboveground litter was replaced on the top of the PVC cylinders for *in-situ* incubation.

### Soil sampling and analysis

At each subplot, three replicate soil samples were collected from PVC cylinders from April 2012 to April 2013 by removing one cylinder at each time, with an interval of 60, 120, 180, 240, and 360 days, separately. Soil horizons included in the cores were simply A horizon. When soil cores were collected, stones and coarse roots were removed from the soil. The samples were stored in cooling boxes and transported to the laboratory, where soil samples were homogenized with 5 mm-sized mesh sieves. One-third of the homogenized samples were frozen at -20°C for microbial phospholipid fatty acid (PLFA) analysis while the remaining portions were air-dried, sieved through 2 mm mesh, and stored at +4°C (<48 h) for other chemical analyses.

### Basic physical and chemical characteristic analyses

Soil temperature at 5 cm depth was measured with a mercury thermometer (1/20°C). Soil moisture was measured by oven drying the soil at 105°C for 24 h and measuring the weight loss. Soil pH was measured using a soil suspension extracted at a 1:2.5 (w/w) soil:water ratio. Soil bulk density was measured using the core method [45]. Soil organic carbon was determined via wet oxidation using dichromate in an acid medium, followed by the FeSO_4_ titration method [46]. Total N was measured by Kjeldahl digestion and distillation azotometry [47].

### Inorganic N and mineralization rate analyses

Soil was extracted with 1 *M* of KCl and inorganic N (NH_4_^+^-N, NO_3_^−^-N and NO_2_^−^-N) was determined by measuring extracts with an automated Continuous-Flow Auto Analyzer (Bran Luebbe AA3, German). Net N ammonification and nitrification rates were calculated from the differences of soil NH_4_^+^ and NO_3_^−^ concentrations between days 0, 60, 120, 180, 240, and 360. Mineral N (N_min_) was calculated by summing the concentration of NO_3-_-N and NH_4_^+^-N. Net mineralization was calculated from the difference of soil inorganic N (NH_4_^+^-N and NO_3-_-N) before and after incubation in the PVC core.

### MBN and SON analyses

Soil microbial biomass nitrogen (MBN) was measured using the chloroform fumigation–extraction method [48]. Soil samples subjected to fumigation and non-fumigation treatments were extracted in 0.5 *M* of K_2_SO_4_ at a ratio of 1:4. The concentration of K_2_SO_4_–extracted total N was analyzed using a modified method of alkaline persulfate oxidation [49] and nitrate was determined by ultraviolet spectrophotometry analysis in a spectrophotometer (Hitachi, UV2300) at 220 and 275 nm. Microbial biomass nitrogen was calculated using a K_*EN*_ factor of 0.45 [48]. Total soluble N was determined by the extracts of non-fumigation soil samples. Soluble organic nitrogen (SON) was calculated by subtracting the concentration of inorganic N from total soluble N.

### Soil hydrolyzable N fractions analyses

Total soil hydrolyzable N (HTN) and its components were analyzed according to the method from Bremner (1965) [50]. In brief, total soil acid-hydrolyzable N was fractionated by mixing 5 mL of 6 *M* of HCl hydrolysis and 2 mL of 5 *M* H_2_SO_4_ in Kjeldahl bottles. In addition, the following three acid hydrolysis solutions of each soil were prepared and analyzed: acid-hydrolyzable ammonium-N (HAN) by adding 2.5 mL of 3.5% MgO to 10 mL of acid hydrolysis solution; acid-hydrolyzable amino sugar N (HASN) by mixing phosphate–borate buffer (pH 11.2) and acid hydrolysis solution in 1:1 ratio (v/v); and acid-hydrolyzable amino acid-N (HAAN) by mixing acid hydrolysis solution and 0.5 *M* NaOH at a ratio of 5:1 (v/v). Then, the nitrogen concentrations in all of these treated solutions were determined by an automatic azotometer with a blank test conducted synchronously. Acid- unhydrolyzable N (UHN) was calculated by subtracting HTN from total N.

### PLFA analyses

Soil phospholipid fatty acids (PLFAs) were extracted and analyzed in triplicate using the modified method of Frostegård and Bååth (1996) [51]. In brief, the lipids were extracted from 3 g of soils with a buffer of chloroform/methanol/citrate mixture at a ratio of 1:2:0.8. Then, neutral lipids, glycolipids, and phospholipids were separated by sequential elution from a silica-bonded solid-phase extraction column (Supelco Silica Tube, 3 mL, 500 mg) using chloroform, acetone, and methanol into distinct layers on a silicic acid column. Afterward, phospholipids were subjected to mild alkaline methanolysis. Phospholipid fatty acids methyl esters were separated on GC/MS (Trace GC Ultra/DSQ II, Thermo Fisher Scientific, Waltham, MA, USA) equipped with a splitless inlet, a BP-5MS column (30 m × 0.25 mm inner diameter and 0.25 μm film thickness). The initial temperature program was 70°C for 2 min and increased to 280°C at 3°C min^−1^. An internal standard of methyl non-adecanoate fatty acid (19:0) was added to quantify peak areas. Individual PLFAs were identified using fatty acid methyl ester standard compounds (Bacterial Acid Methyl Esters Mix; Supelco, Bellefonte, PA). For characterizing community structure, individual fatty acids are used as signatures for various functional groups of microorganisms [52–54]. In particular, bacterial biomass (PLFA_Bact_) was quantified as the sum of the i14:0, a15:0, i15:0, 15:0, i16:0, a16:0, 16:0, 16:1ω9c, a17:, i17:0, 17:0, cy17:0, 10Me18:0, and cy19:0 fatty acids. Fatty acid peaks of i14:0, a15:0, i15:0, i16:0, a17:0, and i17:0 were used as indicators for Gram-positive bacteria and the peaks of 16:1ω9c, cy17:0, and cy19:0 were used for Gram-negative bacteria. Furthermore, 18:1ω9c and 18:2ω9, 12c were used as fungal PLFA markers. As for actinomycetes peak, we used 10Me18:0, and the sum of all PLFA peaks were used to estimate the total microbial biomass.

### Statistical analysis

The differences in soil N and microbial parameters over the period of six months were compared by ANOVA followed by Duncan's post hoc test at a significance level of 5%. An independent two-sample *t*-test was further used to examine the differences within a single parameter of two different forested soils at a single time point/month of sampling. All figures were made by Origin 8.5 software for soil N fractions and microbial communities. A canonical correlation analysis (CCA) was used to evaluate the relationship between soil N fractions and soil microbial indicators. The 12 indices of the canonical variates for soil N (N-CV) were: acid HTN (N1), acid hydrolyzable ammonium (N2), acid HAAN (N3), acid HASN (N4), acid hydrolyzable unknown N (HUN) (N5), acid-non-hydrolyzable N (N6), NH_4_^+^-N (N7), NO_3_^−^-N (N8), NO_3_^−^-N (N9), ON (N10), SON (N11), and MBN (N12). The six indices of the canonical variates for soil microbes (M-CV) were: total PLFAs (P1), bacterial all (P2), Gram-positive bacteria (P3), Gram-negative bacteria (P4), fungi (P5), and actinomycetes (P6). To determine how microbial PLFAs were related to different soil N forms, soil N groups were split into two the sub-groups, namely, the total soil hydrolyzable N components (N1, N2, N3, N4, N5 and N6) and the rest N fractions (N7, N8, N9, N10, N11 and N12). Then, the two groups of soil N indicators were separately subjected to CCA with the PLFA data.

## Results

### Physical and chemical soil characteristics

In the SA and PdF soils, temperature at 5 cm depth in the soil varied between 1.24°C in December and 25.12°C in August (Table 2). Soil moisture ranged from 7% to 20%. Averaged soil moisture was higher in SA soil than that in PdF soil. By contrast, the bulk density of PdF was higher than that of the SA. For each soil sample, pH did not differ significantly within those months (Table 2). By comparing the two-time points of April 2012 and April 2013, contents of SOC significantly increased by 4.7% and 6.0% in SA and PdF; soil C/N ratio showed a significant increase of 12.0% and 30.4% in SA and PdF, respectively.

**Table 2:** Soil basic physical and chemical properties.

	Month	SOC	C:N	pH	Soil moisture	Bulk density	Rainfall	Soil temperature
		(g·kg^−1^)	ratio		(%)	(g·cm^−3^)	(mm)	(°C)
	Apr. (2012)	9.50±1.24b	10.4±2.2b	8.22±0.46a	14±0.7a	1.27±0.04a	20	2.54±1.11d
	Jun. (2012)	7.89±1.45c	10.4±2.3b	8.36±0.42a	7±0.3c	1.25±0.01a	75	16.72±4.21b
**PdF**	Aug. (2012)	6.64±2.05d	9.0±0.8c	8.34±0.36a	7±0.2c	1.27±0.04a	110	24.31±9.89a
	Oct. (2012)	7.20±0.32c	9.6±2.0c	8.63±0.42a	9±0.9b	1.28±0.02a	48	6.68±2.23c
	Dec. (2012)	7.42±1.31c	9.5±1.1c	8.44±0.47a	12±0.7a	1.27±0.07a	15	1.24±1.00e
	Apr. (2013)	10.07±1.52a	13.5±1.2a	8.38±0.06a	10±0.3a	1.29±0.09a	21	3.81±2.23d
	Apr. (2012)	9.84±0.17b	9.9±1.0b	8.20±0.17a	19±0.4a	1.06±0.04a	24	3.32±1.01d
	Jun. (2012)	9.04±1.36d	8.9±1.1c	8.33±0.86a	14±1.0b	1.09±0.03a	81	14.18±4.23b
**SA**	Aug. (2012)	9.12±0.36c	9.8±1.0b	8.44±0.48a	11±0.5b	1.06±0.12a	117	25.12±5.39a
	Oct. (2012)	10.19±1.86a	11.2±0.4a	8.20±1.95a	20±6.5a	1.06±0.01a	55	5.57±2.19c
	Dec. (2012)	9.51±1.91b	9.7±1.2b	8.61±0.56a	18±5.6a	1.09±0.02a	20	1.79±0.89e
	Apr. (2013)	10.31±0.74a	11.1±1.2a	8.35±0.69a	13±0.2b	1.09±0.04a	20	3.11±2.23d

SA (*Sibirica apricot*), PdF (*P*. *davidiana Franch*).

Data are means ± SE (standard error), n = 6. For each plant species, different lowercase letters indicate statistical difference among the 6 months at 0.05 level by ANOVA, followed by Duncan post-hoc.

### Inorganic N

**Fig 1:**
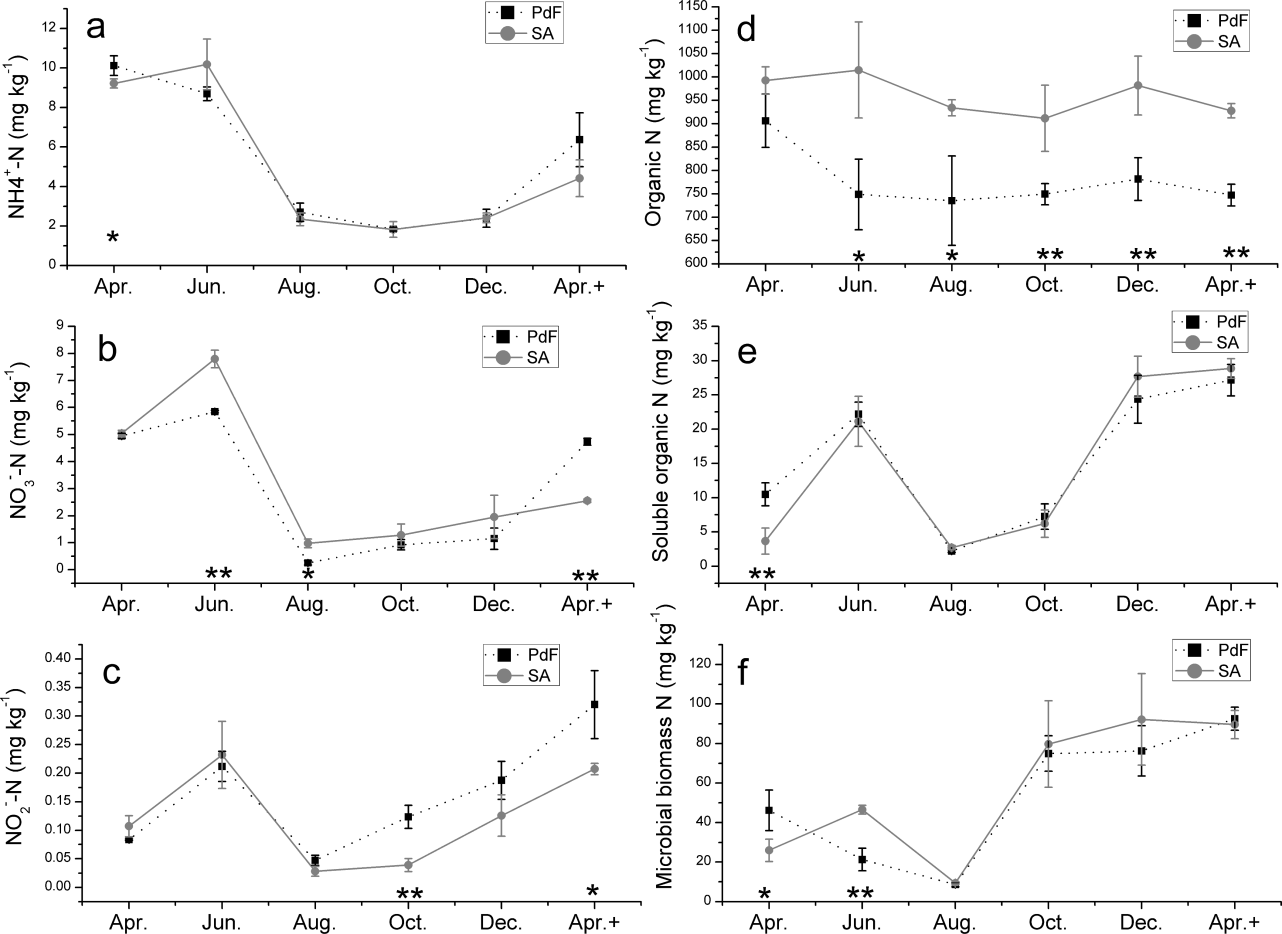
Bimonthly changes in soil N concentrations in the *Sibirica Apricot* (SA) and *Prunus davidiana Franch* (PdF) soils within one year.

The concentration of three inorganic N species (NH_4_^+^-N, NO_3_^**−**^-N, and NO_2_^**−**^-N) was the highest in June and the lowest in August (NO_3_^**−**^-N, and NO_2_^**−**^-N) and October (NH_4_^+^-N; Fig 1A, 1B and 1C) in both soils. Significant difference in inorganic N between the two soils varied within a month (that is, in April (NH_4_^+^-N), in June and August (NO_3_^**−**^-N)), in October (NO_2_^**−**^-N). In comparison with the start of the experiment (from April to June), NH_4_^+^-N concentration increased by 10.5% in SA and contrastingly decreased by 14.1% in the PdF; NO_3_^**−**^-N concentration increased by 54.8% and 18.1% in SA and PdF, respectively; NO_2_^**−**^-N concentration increased 1.2 and 1.5 times in SA and PdF respectively. By comparison, apparent decreases were observed for all three forms of inorganic N in the two soils after June. Specifically, NH_4_^+^-N concentrations decreased by ~80% until October. NO_3_^**−**^-N and NO_2_^**−**^-N concentrations decreased by ~85% and ~55% in August.

### Ammonification, mineralization and nitrification

Average annual rates of ammonification, nitrification and mineralization were similar between the two soils, whereas the three rates varied strongly with time (Table 3). During the first incubation period (0–60 days, from April to June 2012), the ammonification rate was 0.016 mg kg^−1^ day^−1^ in the SA soil and −0.024 mg kg^−1^ day^−1^ in the PdF soil (Table 3). The nitrification rate was three times higher in SA (0.046 mg kg^−1^ day^−1^) than in PdF (0.015 mg kg^−1^ day^−1^) (P < 0.05). The mineralization rates were 0.062 mg kg^−1^ day^−1^ in SA soil and −0.009 mg kg^−1^ day^−1^ in PdF soil. During the days of 60–120 (from June to August), negative rates were observed for the ammonification (−0.13 mg kg^−1^ day^−1^), nitrification (−0.114 mg kg^−1^ day^−1^) and mineralization (−0.244 mg kg^−1^ day^−1^), with a relatively higher incidence of these rates in SA soil compared with PdF soil (Table 3). No significant changes in inorganic N transformation rates were noted from days 120−180 (from August to October). However, the rates of nitrification and mineralization were significantly higher in SA (0.012 and 0.021 mg kg^−1^ day^−1^) than in PdF (0.004 and 0.013 mg kg^−1^ d^−1^) during days 180−240 (from October to December). During days 240−360 of experiment (from December 2012 to April 2013), PdF presented higher ammonification, nitrification and mineralization rates (0.066, 0.060, and 0.043 mg kg^−1^ day^−1^, respectively) compared with SA (0.033, 0.010, and 0.043 mg kg^−1^ day^−1^, respectively; Table 3).

**Table 3:** Ammonification, mineralization, and nitrification rates (mg kg^−1^ d^−1^) in SA and PdF soils along temporal patterns.

Time interval	Ammonification		Nitrification		Mineralization	
	SA	PdF	SA	PdF	SA	PdF
Apr−Jun	**0.016 a**	−0.024 b	**0.046 a**	**0.015 b**	**0.062 a**	−0.009 b
Jun−Aug	−0.131 a	−0.100 a	−0.114 a	−0.093 a	−0.244 a	−0.193 a
Aug−Oct	−0.009 a	−0.014 a	**0.006 a**	**0.011 a**	−0.004 a	−0.003 a
Oct−Dec	**0.010 a**	**0.009 a**	**0.011 a**	**0.004 b**	**0.021 a**	**0.013 b**
Dec−Apr+	**0.033 b**	**0.066 a**	**0.010 b**	**0.060 a**	**0.043 b**	**0.126 a**
Annual mean	−0.013 a	−0.014 a	−0.008 a	−0.007 a	−0.024 a	−0.023 a

Positive values are shown in bold. Different lowercase letters (between SA and PdF) indicate independent samples *t*-test at a significance level of 0.05. “April+” means April in the year of 2013 and the other months were all in 2012.

### ON, SON, and MBN

Organic nitrogen (ON) concentration remained stable over the year for each soil (Fig 1D). Annual average ON concentration was higher in SA (960 mg kg^−1^) soil than that in PdF (770 mg kg^-1^) soil. The concentrations of SON and MBN varied similarly (Fig 1E and 1F). After 2 months of incubation from April to June, the SON concentrations increased by approximately fivefold and onefold in SA and PdF, respectively (Fig 1E). Microbial biomass nitrogen concentration increased by 130.5% in SA and decreased by 40.7% in PdF (Fig 1F).

The lowest values were observed after 4 months of incubation in August (Fig 1E and 1F); SON and MBN concentrations decreased by 25.6% and 79.1% in SA and by 54.4% and 75.8% in PdF, respectively. After this time point, the concentrations all increased continuously with a strong MBN trend. At the end of the incubation trial in April 2013, the content of MBN and SON increased by ~7 and >3 times, respectively (Fig 1E and 1F). The averaged concentrations of MBN (calculated on an annual base) and SON were approximately 55 and 15 mg kg^−1^, and no significant difference can be detected between the two soils.

### Hydrolyzable total N (HTN) and its components

**Fig 2:**
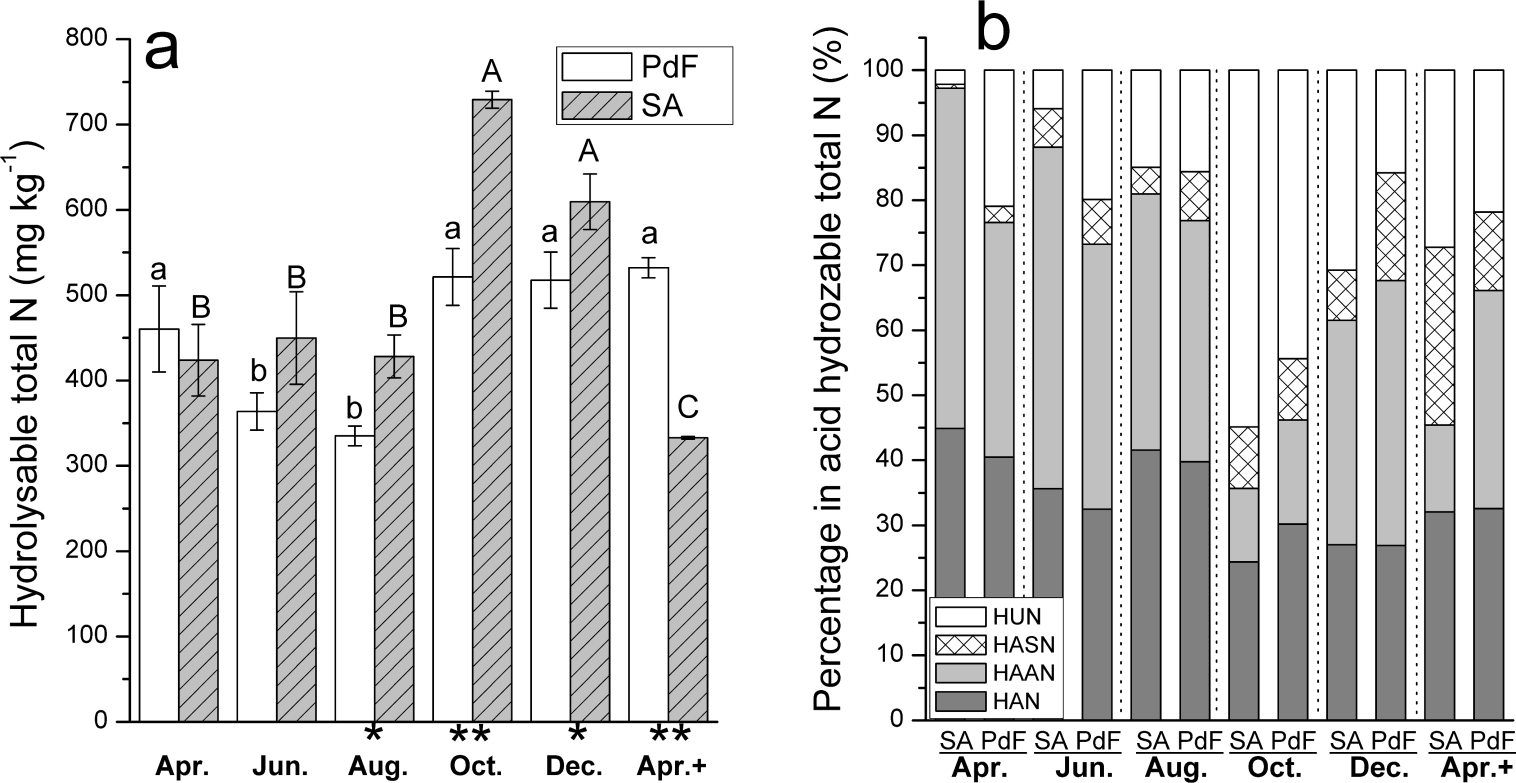
Bimonthly changes of the HTN in SA and PdF soils.

Hydrolysable total N (HTN) content significantly increased (p *<* 0.05) from August to October, and significant difference was observed from August to December (p < 0.05; Fig 2A) in both soils. In comparing monthly change of HTN between the two soils, PdF showed a stable change with no significant difference (p > 0.05) in April, October, December 2012, and April 2013. Hydrolysable total N content of SA was significantly higher (p < 0.05) in October and December than that in other months (Fig 2A).

For hydrolysable total N components, HAAN was dominant except for the months of October 2012 and April 2013. In October, the two soils showed highly significant difference (p < 0.01) for HTN content (Fig 2A) whereas the relative percentage of HTN components was similar: the highest percentage of HUN (~50%), followed by HAN, HAAN, and HASN (Fig 2B). By comparison, from April to August, HTN was dominated by the fractions of the HAAN and HAN in both soils. However, the fractions of HUN and HASN gradually increased in the subsequent months (Fig 2B). In the course of a year (from April of 2002 to 2003), within the HTN fraction, soil HSAN exhibited a marked increase of 10 and 5 times in SA and PdF, respectively (Fig 2B).

### Change in microbial community structure as determined by PLFA

**Fig 3:**
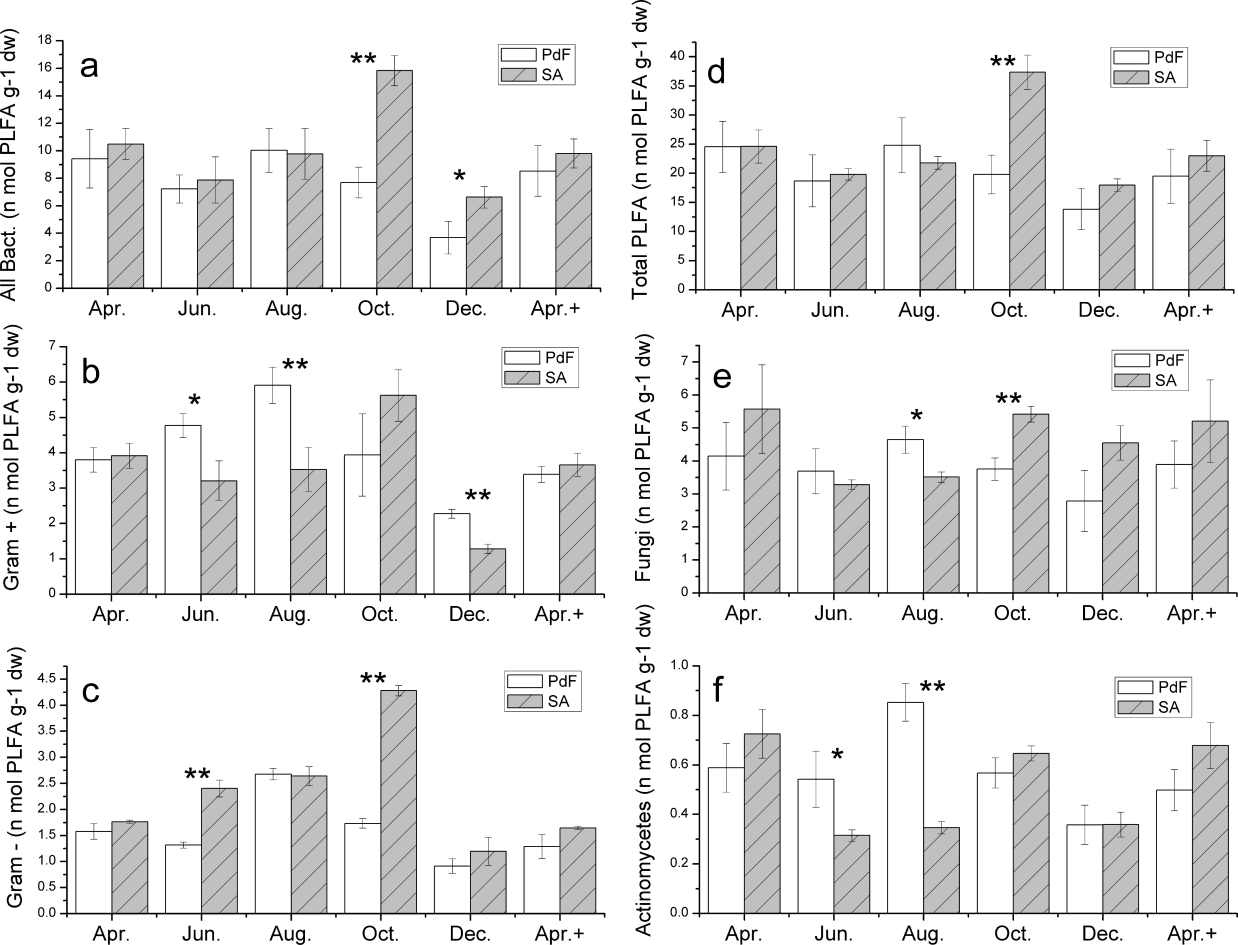
Microbial community structure (determined by PLFAs analysis) of bimonthly variations in SA and PdF soils.

Both soils of SA and PdF were bacteria dominated, with a relative abundance exceeding 60%. No significant plant species effect on microbial PLFA was detected at the start and end of the experiment (Fig 3). However, in warm months (June and August), the contents of Gram-positive PLFA and actinomycetes PLFA were significantly higher in PdF soil than those in SA soil (Fig 3B and 3F), whereas in October, the total PLFA and the PLFAs belonging to all bacteria, Gram-negative and fungi, were all significantly higher in SA soil compared with PdF soil (Fig 3).

### Correlation between soil N and microbial indicators

Canonical correlation analysis (CCA) was performed using soil N and microbial PLFA data. In total, six pairs of canonical variates (CVs) were extracted individually (Tables 4 and 5). For the HTN components, the canonical correlation between the first soil N canonical variate (N-CV1) and the first microbial canonical variate (P-CV1) was significant (R = 0.989) and showed a good fit (p = 0.0001). The first CV mainly reflected the relationship between the acid hydrolyzable ammonium (N2) and bacterial PLFA (P2). Approximately 43% of the variance in P-CV1 was explained by the N-CV1 (as indicated by the proportion that explained from between-cluster; Table 4). The contributions (evaluated by the absolute value of canonical coefficient) of the different forms of acid hydrolyzable N as evaluated by the canonical coefficient of CV were in the order of HAN > HAAN > HASN > unknown N > UHN > HTN. By contrast, microbial PLFA contributions were in the order of bacteria > Gram (−) > fungi > Gram (+) > total PLFA > Actinomycetes.

**Table 4:** Canonical correlation coefficients between ON components and PLFAs.

Canonical correlation coefficient significance test					Proportion that can be explained (%)			
					P-CV		N-CV	
No.	Correlation	Chi-SQ	DF	Sig.	Within-	Between-	Within-	Between-
					cluster	cluster	cluster	cluster
1	0.989	174.28	36	0.0001	45.8	43.2	38.1	37.6
2	0.954	17.03	25	0.880	10.5	9.5	17.8	16.2
3	0.801	6.23	16	0.985	30.7	26.1	8.5	5.5
4	0.519	1.69	9	0.996	5.5	1.5	14.6	3.9
5	0.182	0.27	4	0.995	7.3	0.2	16.8	1.2
6	0.111	0.055	1	0.814	10.2	0.1	14.3	0.2
U_1_ = 0.137N_1_ − **1.345N**_**2**_ + 0.919N_3_ − 0.820N_4_ − 0.559N_5_ − 0.506N_6_								
V_1_ = −0.322P_1_ − **2.847P**_**2**_ + 0.545P_3_ + 1.492P_4_ +1.320P_5_ − 0.315P_6_								

CCA was performed using the soil N (ON components) and PLFAs; six pairs of canonical variates (CVs) were extracted (as shown by the numbers in the first column). U1 and V1 refer to the first group equation between ON components (N-CV) and the PLFA canonical variate (P-CV), which present the highest significant coefficient of 0.0001. The remaining four equations of U2,V2 − U5,V5 did not appear because their canonical correlation coefficients were higher than 0.05. The indices of N-CVs and P-CVs were as follows: Acid-hydrolyzable TN (N1), acid-hydrolyzable ammonium (N2), acid hydrolyzable amino acid N (N3) acid-hydrolyzable amino sugar N (N4), acid-hydrolyzable unknown N (N5), and acid non-hydrolyzable N (N6), as well as total PLFAs (P1), bacterial all (P2), Gram-positive bacteria (P3), Gram-negative bacteria (P 4), fungi (P 5), and actinomycetes (P 6).

**Table 5:** Canonical correlation coefficients between soil N fractions and PLFAs.

Canonical correlation coefficient significance test					Proportion that can be explained (%)			
					P-CV		N-CV	
No.	Correlation	Chi-SQ	DF	Sig.	Within-	Between-	Within-	Between-
					cluster	cluster	cluster	cluster
1	0.994	169.407	36	0.0001	53	48.8	44.8	42.7
2	0.974	22.516	25	0.606	17.2	16.3	12.8	12.1
3	0.782	9.214	16	0.904	16.7	10.2	4.1	2.5
4	0.753	4.963	9	0.837	17.1	9.7	32.6	24.2
5	0.442	1.193	4	0.879	20.9	4.1	4.9	1
6	0.215	0.214	1	0.644	25.2	1.2	30.9	1.4
N_1_ = **3.427 N**_**7**_ − 2.998N_8_ − 0.257N_9_ + 0.002N_10_ + 0.048N_11_ + 1.246N_12_								
V_1_ = **2.565P**_**1**_ + 0.018P_2_ − 1.967P_3_ − 1.147P_4_ − 1.873P_5_ + 1.933P_6_								

CCA was performed using the soil N fractions and PLFAs; six pairs of canonical variates (CVs) were extracted (as shown by the numbers in the first column). U1 and V1 refer to the first group equation between soil N (N-CV) and the PLFAs canonical variate (P-CV), which presents the highest significant coefficient of 0.0001. The remaining four equations of U2, V2 − U5, V5 did not show because its canonical correlation coefficients were higher than 0.05. The indices of N-CVs and P-CVs were NH4+-N (N7), NO3−-N (N8), NO3−-N (N9), ON (N10), SON (N11), SMN (N12), as well as total PLFAs (P1), bacterial all (P2), Gram-positive bacteria (P3), Gram-negative bacteria (P4), fungi (P5), and actinomycetes (P6)

As for the inorganic N and other ON fractions, the canonical correlation between the first soil N CV (N-CV1) and the first PLFA canonical variate (P-CV1) was significant (R = 0.994) with a favorable fit (p = 0.0001). The first CV mainly reflected the relationship between the NH_4_^+^-N (N2) and the total PLFA (P2). Approximately 50% of the variance in the P-CV1 was explained by the N-CV1 (Table 5; as shown by the “between-cluster” percentage). The contributions of soil N fractions, as evaluated by the canonical coefficient of CV, were in the order of NH_4_^+^-N > NO_3_^−^-N > MBN > NO_2-_-N > SON > ON. By contrast, microbial PLFA contributions were in the order of total PLFA > Gram-positive > actinomycetes > fungi > Gram-negative > bacteria.

## Discussion

### Influence of plant species

Plant species influence soil nutrient availability through their effects on litter decomposition, nutrient uptake, inputs, and losses [55,56]. *Sibirica Apricot* (SA) and *Prunus davidiana Franch* (PdF) are two typical revegatation types planted on the Loess Plateau in China to reduce the rate of soil erosion. Our results showed the stronger effects of variations in plant species on soil ON than that in soil inorganic N stocks, which is further in line with our first hypothesis. The average concentrations of soil NH_4_^+^-N did not differ significantly between the SA and PdF. This finding agrees with those of Ren et al. (2011), who reported that NH_4_^+^-N concentration was unvaried even under different vegetation types among coniferous, mixed, and broadleaf forests. However, this result contradicts previous findings [57,58] where site specific characters, such as soil and bed rock type, temperature, moisture, and vegetation performed a key role in modifying N stocks in forest soils. *Sibirica Apricot* soil showed higher ON content than PdF. This finding may be attribute to inherent soil/site difference and plant species. However, given the similar land-use history and soil type of the two sites, we mainly ascribe the reason of increased accumulation of N in soils to aboveground leaf litter traits and its decomposition rates. Lignin is considered as a major recalcitrant part of leaf litter and is found to be relatively higher in SA (14%) than that in PdF (12%). This may further lead to increased accumulation of N in SA soil since high lignin content in leaf litter inhibits the mineralization of organic N fractions in most cases [59–61]. For hydrolysable total N components, a stable variation in amino acid concentration was detected, as is consistent with a study performed by Jones et al. (2009), at a global scale that covered 40 sites [62]. The steady change of amino acid was probably due to the fact that it is the major component of slowly-decomposing organic N. Moreover, the decomposing process that converts high weight organic matter (such as humus and lignin fractions) into low weight matters (such as amino acid and amino sugar), is the rate-limiting step during soil organic matter decomposition. Differences in aboveground vegetation reportedly affect soil microbial communities [63,64]. Our results showed that high relative abundance of microbial groups exist in the soils with high relative percentage of HASN (Fig 2) and N_min_. A possible connection between the N fractions and the microbes is the high relative abundance of microbial groups, which can result in high microbial activities, microbial metabolism, and ultimately high microbial residues (which can be marked by HASN; [10]). This increment can further accelerate soil N mineralization (resulting in increased content of N_min_); therefore, UHN fractions, such as plant lignin fractions and/or dominant structural components of humic compounds, are correspondingly high [65].

In summary, the comparison between two sites planted with different plant species showed minor changes in soil microbial community and soil N (Table 3 and Fig 3). Therefore, we expected that the real differences were induced by temporal effects.

### Temporal effects on soil N and microbial community

This analysis suggests that in comparison with variations in plant species, temporal variations result in additional changes in soil microbial communities and N contents, which support the second hypothesis of our study. Therefore, we further focused on whether soil measurement varies synchronously with time. Given that monthly-based changes are mainly reflected by the changes in abiotic factors, such as temperature, soil moisture, and rainfall, we compared the measured soil parameters and abiotic factors. The lowest soil temperature (~1.5°C) was observed in December 2012 (winter) with SA and PdF treatment, whereas, in spring, the temperature increased up to 3.5°C in April 2013 (Table 2). Meanwhile, the changes of microbial biomass and abundances of all microbial groups were synchronous with time, lowest values in December and increased in the following year (Fig 3). Temperature-related microbial shifts during the winter–spring transition are common features across various systems, and reveals a widespread biogeochemical pattern in seasonally frozen ecosystems [66]. In our study, temporal variations from December 2012 to April 2013 (accompanied by seasonal freeze-thaw transitions) were followed by non-synchronous changes in microbial biomass and soil N contents. We observed a general decrease in microbial biomass and an increase in large parts of the contents of inorganic N and SON, which are related to the acceleration of the release of soil N components at higher temperature; by contrast, soil microorganisms tend to lag behind when available nutrients are incorporated into their biomass to maintain their self-metabolism [67,68]. In general, temporal variations of individual microbial groups (determined by PLFAs analysis) were synchronous regardless of the differences in plant species (Fig 3). This result agrees with those of Liu et al. (2016), who observed that microbial communities are strongly affected by environmental constraints, such as sampling time, soil moisture, and air temperature [69]. In a similar study conducted on the soils of tall birch (*Betula glandulosa*) and surrounding dwarf birch hummock vegetation, the fungal dominance, principal fungal, and bacterial types also exhibited synchronous variation with time [27].

In comparison with microbial groups, soil N fractions varied non-synchronously. The concentrations of NO_2_^−^-N, MBN and SON were the highest at the end of the experiment, whereas the concentrations of NH_4_^+^-N, NO_3_^−^-N and ON were the highest at the initial period from April 2012 to June 2012. The lowest rates of net ammonification, nitrification, and mineralization were found from June 2012 to August 2012 (Table 3). These results were comparable with those of a study considering different vegetation types in northern China[70]. From June to August, the high soil C:N ratios (>9; Table 2) coupled with high soil rainfall (averaged 95 mm; Table 2) and moderate temperature (15–25°C; Table 2) possibly promoted net N immobilization in the form of amino acid N because the highest relative percentage of the HAAN during the same period was detected (Fig 2). This finding is consistent and supported by results from other studies showing that HAAN is closely associated with microbial metabolism and functions as an important storage pool for immobilized N [10,71,72].

### Relationship between microbial populations and N

Considering that soil N mineralization is a biological process driven by microbial activity, we tracked the changes in soil microbial parameters accompanied by N mineralization processes via *in-situ* buried soil core method. This method has been used as a common method for estimating N mineralization rates in soils [73,74]. From a microbial perspective, the *in-situ* core method exerts certain influences soil microbes due to the absence of C input above- and belowground. For instance, as a result of top-sealing the cores (with the purpose of preventing N deposition and leaching loss), external C-substrate availability becomes a limiting factor for soil microbial communities [75] because the quantity and chemical composition of aboveground leaf litter play an important role in shaping microbial community structure in forest soils [69,76]. At the early stages of litter decomposition, increased input of leaf litter-dissolved organic matter favors the growth of bacteria over that of fungi [77,78]. Therefore, in comparison to the soil inside the core, surrounding soil tends to harbor high amounts of bacterial biomass. Given that this method is equivalent to root exclusion treatments (which eliminate C inputs except via capillary flow through the bottom cotton), the soil core approach decreases fungal biomass and alters the bacterial community structure in forest soils [79–81].

Although absence of roots and leaf litter induced by the soil core method may lead to a decrease in fungal and bacterial biomass, this method remains reliable in maintaining similar soil microenvironment for soil microbes during *in-situ* incubation [73,74]. Moreover, the possible differences from the method itself can be treated as systematic errors and are largely ignored given the following considerations: i) the two forested soils were subjected to the same (same PVC cylinder material, buried at the same depth) soil core incubation; and ii) the main aims of our experiment were not to compare the changes of soil microbial communities inside and outside of the core but to determine their temporal variations and relationships with soil N fractions. In view of the temporal patterns of soil N and microbial community, we aimed to associate measured soil N and microbial variables by using CCA. The identified relationship was in line with our third hypothesis. Canonical correlation analysis showed the relationship between the NH_4_^+^-N and the total PLFAs, which were the most sensitive indicators related to microbially-mediated soil N variation (Table 5). Given that the total PLFA generally comprises the total biomass of living cells, the identified relationship highlights the link among all living microbes and easily available concentration of NH_4_^+^-N.

Owing to the non-synchronous change in soil N fractions (as discussed before), the mechanisms by which soil organic N components interact with individual microbial groups should be clarified. The relationship between ON components and microbial PLFAs were indicated by the link between bacterial PLFAs and the easily available form of HAN (Table 4). For the N source, a large fraction of soil HAN (contributes about 20–35%) was derived from acid-labile organic constituents, such as exchangeable and clay-fixed NH_4_^+^ [2,3]. Therefore, soil bacterial groups are possibly prone to utilizing N on labile substrates. Bell et al. (2008) found that seasonal and annual variability of soil bacterial activity was the most closely associated with extractable NH_4_^+^-N, pH and SOM. However, the mechanism of how bacterial groups interact with various NH_4_^+^ forms cannot be clearly uncovered by the present study because NH_4_^+^-N production rates were correlated positively with large pools and production rates of dissolved soil C and N, high quality litter inputs, and low soil C concentration [27]. Tahovská et al. (2013) also proposed that the structure of the bacterial community was related to the dissolved organic C and the concentrations of C and N in microbial biomass [82]. We expect that future research, combined with not only soil N but also C and easily available fractions, will provide further understanding of this relationship. In summary, we used the multivariate analysis of CCA to clarify the main link during a microbially-mediated N change. However, we need to carefully explain the two relationships. The obtained links help us to focus on the main features of soil N–microbes variation, but these findings does not simply mean that bacteria were closely correlated with organic HAN and that the easily available concentration of NH_4_^+^-N is preferred by all living microbes.

## Conclusions

To improve our understanding of soil N and microbial changes on a broad forested region in the Chinese Loess Plateau, we selected two commonly cultivated forests sites as representative ecological systems and explored the relationship between soil N fractions and microbial groups during *in-situ* N mineralization. A comprehensive investigation on soil N fractions revealed that the total ON (accounting for the highest percentage within soil N) exhibited a minor temporal change, whereas the other forms of N exhibited a non-synchronous variation with time. As a microbially-mediated process, the dynamics of different soil microbial groups were more affected by time than by plant species. Our data highlighted the importance of total PLFAs, microbial PLFAs belonged bacteria, and easily-accessible inorganic NH_4_^+^-N and organic HAN in understating the main links between soil N fractions and microbial groups.

## Supporting information

S1 Table The original date of nitrogen fractions changes. (XLSX)

S2 Table The changes of components of soil organic nitrogen. (XLSX)

S3 Table The changes of soil PLFA. (XLSX)
